# Applying the PRECEDE-PROCEED model to develop MommaConnect: a digital healthcare platform for addressing postpartum depression and improving infant well-being

**DOI:** 10.37349/en.2024.00052

**Published:** 2024-08-06

**Authors:** Bobbie Posmontier, June Andrews Horowitz, Pamela A. Geller, Mona Elgohail, Mary McDonough, Kayla Alvares, Jaleesa Smoot, Katie Chang, Tony Ma

**Affiliations:** 1 Jefferson College of Nursing, Thomas Jefferson University, Philadelphia, PA 19107, USA; 2 College of Nursing & Health Sciences, University of Massachusetts Dartmouth, Dartmouth, MA 02747, USA; 3 Department of Psychological and Brain Sciences, Drexel University, Philadelphia, PA 19104, USA; 4 Benten Technologies, Manassas, VA 20110, USA

**Keywords:** Postpartum depression, digital healthcare platform, maternal-infant interaction, maternal functioning, PRECEDE-PROCEED model, mobile health application

## Abstract

The PRECEDE-PROCEED model is a comprehensive planning and theoretical framework that incorporates epidemiological, environmental, behavioral, and social factors systematically to design, implement, and evaluate health promotion programs. As such, PRECEDE-PROCEED is a highly effective tool for addressing complex and significant public health concerns like postpartum depression (PPD). PPD negatively impacts mothers and their infants, with studies showing that approximately one in eight mothers experience PPD, leading to adverse effects on maternal functioning and infant development. However, access to specialized evidence-based treatment remains significantly limited due to barriers including social determinants of health. This paper explores the application of the PRECEDE-PROCEED model as a planning and theoretical framework for the design and development of MommaConnect, an innovative digital healthcare platform aimed at reducing PPD symptoms and improving maternal-infant interaction while overcoming barriers to treatment. Key components of the MommaConnect design and development process are mapped onto the steps of the PRECEDE-PROCEED model. MommaConnect features are aligned with specific stages of the model, from assessing, predisposing, enabling, and reinforcing factors to designing, implementing, and evaluating the intervention. By leveraging this model, MommaConnect represents a promising innovative approach to address PPD to improve maternal functioning and infant health in a digitally-enabled era. This paper underscores the importance of utilizing a framework like the PRECEDE-PROCEED model in the design and development of innovative healthcare solutions.

## Introduction

Our research team has been using the PRECEDE-PROCEED model [1, 2] to guide the design and development of MommaConnect, a patient-centered digital healthcare platform that can be accessed on a variety of electronic devices (e.g., smartphone, tablet, computer) to facilitate remote delivery of psychotherapy and psychoeducation by clinicians to women experiencing postpartum depression (PPD). The eight-step PRECEDE-PROCEED model is a comprehensive, participatory, and ecological model that has guided healthcare intervention design and development for health conditions for the past 30 years [1, 2]. The purpose of this paper is to review the PRECEDE-PROCEED model and illustrate how the model has guided the planning and development of MommaConnect to treat PPD and improve mother-infant interaction.

PPD affects approximately one in eight women, with rates doubled among women identifying as low-income, and is known to have numerous adverse effects on maternal functioning and infant well-being [3]. Without treatment, women with PPD are at significant risk for chronic and treatment-resistant depression, poor maternal functioning, adverse family functioning, and suicide [4–8]. PPD also increases the risk for impaired mother-infant interaction, reduced maternal attention to infant safety, decreased breastfeeding, and poor infant developmental outcomes [4–8]. Specifically, infants of mothers who experience PPD are at significant risk for poor long-term cognitive, emotional, and neurobehavioral development [9–11] because of the mothers’ impaired ability to respond sensitively and contingently to their infants [10–15]. Compared to mothers without PPD, mothers with depression may be less affectionate and less engaged with their infants or overly intrusive and less responsive to their infants’ engagement and disengagement cues [16–18]. In the long term, these maternal behaviors are associated with impaired child development (e.g., cognitive, socioemotional) as well as child psychiatric disturbance, including conduct, anxiety, depression, and substance use disorders with a prevalence exceeding 40% [16].

Despite growing treatment options, most women with PPD face significant barriers to receiving care. Only 12–37% of women with PPD bring their depressive symptoms to professional attention and receive treatment in the postpartum period [8–11, 19]. Barriers to treatment may include lack of awareness of signs and symptoms of PPD, stigma surrounding mental illness, fears of having children removed from the home, competing childcare and work responsibilities, and limited geographic accessibility to treatment, including poor access in rural areas. Further, with 3.8 million births per year in the USA [20], there is essentially one intensive outpatient perinatal treatment program per 21,000 women with PPD, further complicating access to quality and specialized care. Only 13 states provide such intensive services, and only three programs specifically address the needs of women with low-incomes and their infants. These women may face even greater challenges to PPD care, including inadequate healthcare coverage, lack of transportation to and from appointments, and living in “medical deserts” with few behavioral healthcare providers who accept Medicaid. Remote treatment options, including telehealth and mobile applications tailored to PPD treatment and improving maternal-infant interaction, may offer reasonable solutions for women who experience poor access to specialized care to prevent and treat adverse maternal and child outcomes.

Our research team is co-creating the MommaConnect digital healthcare platform accessible by smartphones, tablets including iPads, and computers for women across the socioeconomic spectrum and their clinicians to increase treatment access by reducing barriers to PPD treatment. Smartphone ownership, the basis of the patient-facing app of the MommaConnect digital healthcare platform, has increased across all income categories in the USA, including a 71% increase among women with incomes less than $30,000 per year and an 85% increase among those with incomes in the $30,000–$99,999 range [21]. Multiple meta-analyses showed mobile health (mHealth) interventions to be efficacious in delivering treatment to the target population [22–24]. Furthermore, mHealth apps built with offline and online capabilities can be leveraged to address broadband and data access issues and promote access to digital education and care [25–27]. MommaConnect is designed with these features to address potential access challenges for women, including those with low socioeconomic status and those with limited geographic accessibility, such as those living in rural areas.

Tailoring a patient-centered digital healthcare platform such as MommaConnect to facilitate remote psychotherapy delivery is intended to help clinicians engage mothers with PPD via a variety of digital devices, address impaired mother-infant interaction at the point of care, and facilitate shared decision-making between clinician and patient. Further, MommaConnect is intended to advance the quality of care, mitigate barriers to treatment access, improve patient experiences, improve maternal-infant mental health outcomes, and capture patient-generated data to guide therapeutic decisions for women who are experiencing PPD and their infants. For example, daily mood monitoring, a feature of MommaConnect, will provide ongoing self-evaluation data for clinicians to gauge trends toward improvement or increasing symptom severity, and take action as indicated.

## Materials

### PRECEDE-PROCEED model

The PRECEDE-PROCEED model is a planning and theoretical framework that provides an educational diagnosis to inform healthcare intervention development. Originally developed in 1980 by Green et al. [28], the model links causal determinants of health to planning and evaluating behavioral change. Rather than exclusively focusing on implementation, the model stresses planning and designing interventions to meet specific individual- and population-level needs [29, 30]. PRECEDE is an acronym for predisposing, reinforcing, and enabling constructs in educational/environmental diagnosis and evaluation. PROCEED is an acronym for policy, regulatory, and organizational constructs in educational and environmental development [29, 30]. PROCEED was added in 1991 to account for social determinants of health and health inequities that impact health behaviors, as well as to incorporate ecological approaches that account for relationships between people and their environment. Finally, in 2005, the PRECEDE-PROCEED model added a participatory approach to give voice to stakeholders and integrate new knowledge from genetics research [29, 31].

**Figure 1.**
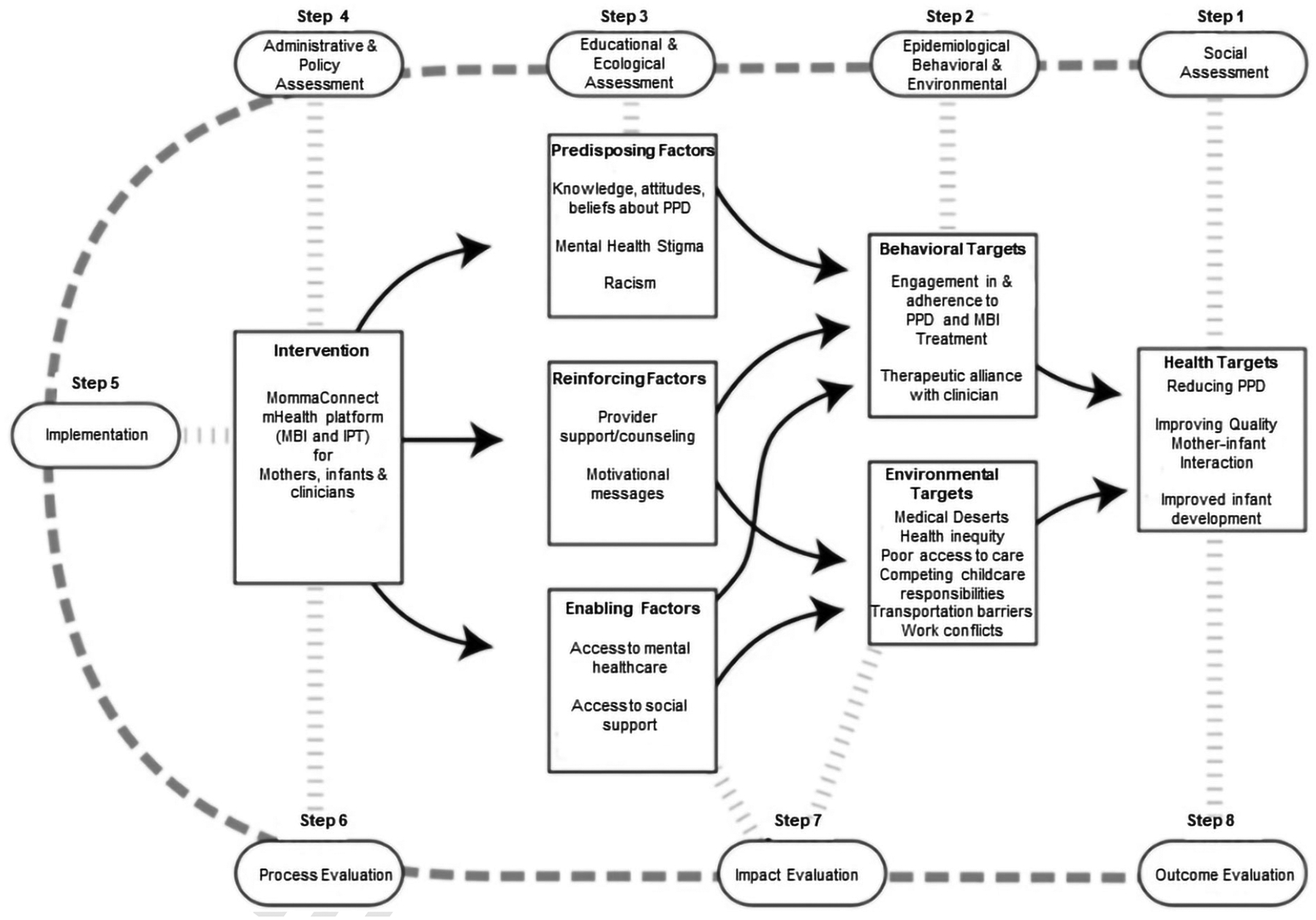
Iterative MommaConnect development process using the PRECEDE-PROCEED model. PPD: postpartum depression; mHealth: mobile health; MBI: Mother-Baby Interaction Therapy; IPT: Interpersonal Psychotherapy [32] *Note*. Adapted with permission from “Development and evaluation of a youth mental health community awareness campaign-the compass strategy” by Wright A, McGorry PD, Harris MG, Jorm AF, Pennell K. BMC Public Health. 2006;6:215 (https://doi.org/10.1186/1471-2458-6-215). CC BY.

The eight-step model comprises four planning and assessment steps, an implementation step, and three evaluation steps (Figure 1).

Specifically, step 1 is comprised of a social assessment using focus groups and interviews to understand community strengths, challenges, and readiness for change. Step 2 is comprised of epidemiological, behavioral, and environmental assessments to identify health-related issues as well as risk factors that influence outcomes. The information gathered from this phase translates priorities into measurable outcomes. Step 3 involves an educational and ecological assessment to identify predisposing, reinforcing, and enabling factors related to the health problem. Step 4 considers organizational and environmental factors as well as individual, family, and peer influences that can affect outcomes to inform health promotion strategies. Finally, steps 5 through 8 address implementation, process, impact, and outcome evaluations. The model allows options for skipping phases as needed and can prioritize important intervention targets.

### Using PRECEDE-PROCEED to develop MommaConnect

Our team chose the eight-step PRECEDE-PROCEED model (Figure 1) to guide the iterative development of the MommaConnect digital healthcare platform because it aligned with the project’s goals. Specifically, the project’s goals were to co-create the MommaConnect digital healthcare platform, including content, and to evaluate the feasibility of MommaConnect with women from diverse backgrounds who had experienced PPD. In addition, we sought consultation from practicing perinatal mental health clinicians and leading researchers in the field on the design and content of the clinician-facing component of the MommaConnect digital healthcare platform.

Although we have not conducted a randomized controlled trial (RCT) to date to measure treatment outcomes formally, to inform the design and development of MommaConnect, we examined program evaluation data among women with PPD from racially and socioeconomically diverse backgrounds in our Mother Baby Connections (MBC) perinatal intensive outpatient program (IOP, Table 1) [33]. Among the women in our program 36 out of 80 (45%) completed the program. There were no significant differences between completers compared to non-completers on age, annual income, insurance type, employment, education, or living with a partner.

We used evidence-based approaches including Mother-Baby Interaction Therapy [34], developed by one of the investigators (June Andrews Horowitz), to address deficits in the mother-infant relationship, in addition to a combination of Interpersonal Psychotherapy, Cognitive Behavioral Therapy, and Dialectal Behavioral Therapy. This combination of therapeutic modalities addresses significant problematic relationships, dysfunctional thinking patterns, and emotional dysregulation contributing to depressive symptoms and sub-optimal maternal functioning. Baseline to exit outcomes measured over a 6-year period showed that mothers, who spent an average of 12 weeks in our treatment program during pregnancy and up to 1 year postpartum, had experienced significant improvements in depression (*p* < 0.001), maternal functioning including the relationship with the infant (*p* < 0.001), dyadic adjustment among partners (*p* < 0.05), parenting stress (*p* < 0.001), perceived stress (*p* < 0.001), emotional regulation (*p* < 0.001), birth trauma (*p* < 0.001), and insomnia (*p* < 0.05), with medium to large effect sizes, as well as high satisfaction with the program (29.4 out of 32) (Table 2).

In addition, before the onset of the COVID-19 pandemic and the design and development of MommaConnect, 4 years of data, showed that women in our program missed 44% (325 out of 728) of their scheduled visits during an average treatment course of 12 weeks. However, after the transition to telehealth delivery, we found that women in the program only missed 11% (18 out of 158) of their scheduled sessions. Reasons for missed visits prior to the pandemic included taking care of a sick child, personal illness, other medical appointments, competing childcare responsibilities, scheduling conflicts, work conflicts, adverse weather conditions, transportation issues, court appointments, and family emergencies. Moreover, our positive MBC outcomes did not drop off when we moved to telehealth delivery. These data provided evidence that the content and telehealth delivery of psychotherapy were appropriate to reduce symptoms of PPD, improve maternal-infant functioning, and provide better access to care. We plan to conduct a future RCT to confirm the findings of our program evaluation data.

## Procedure

**Figure 2.**
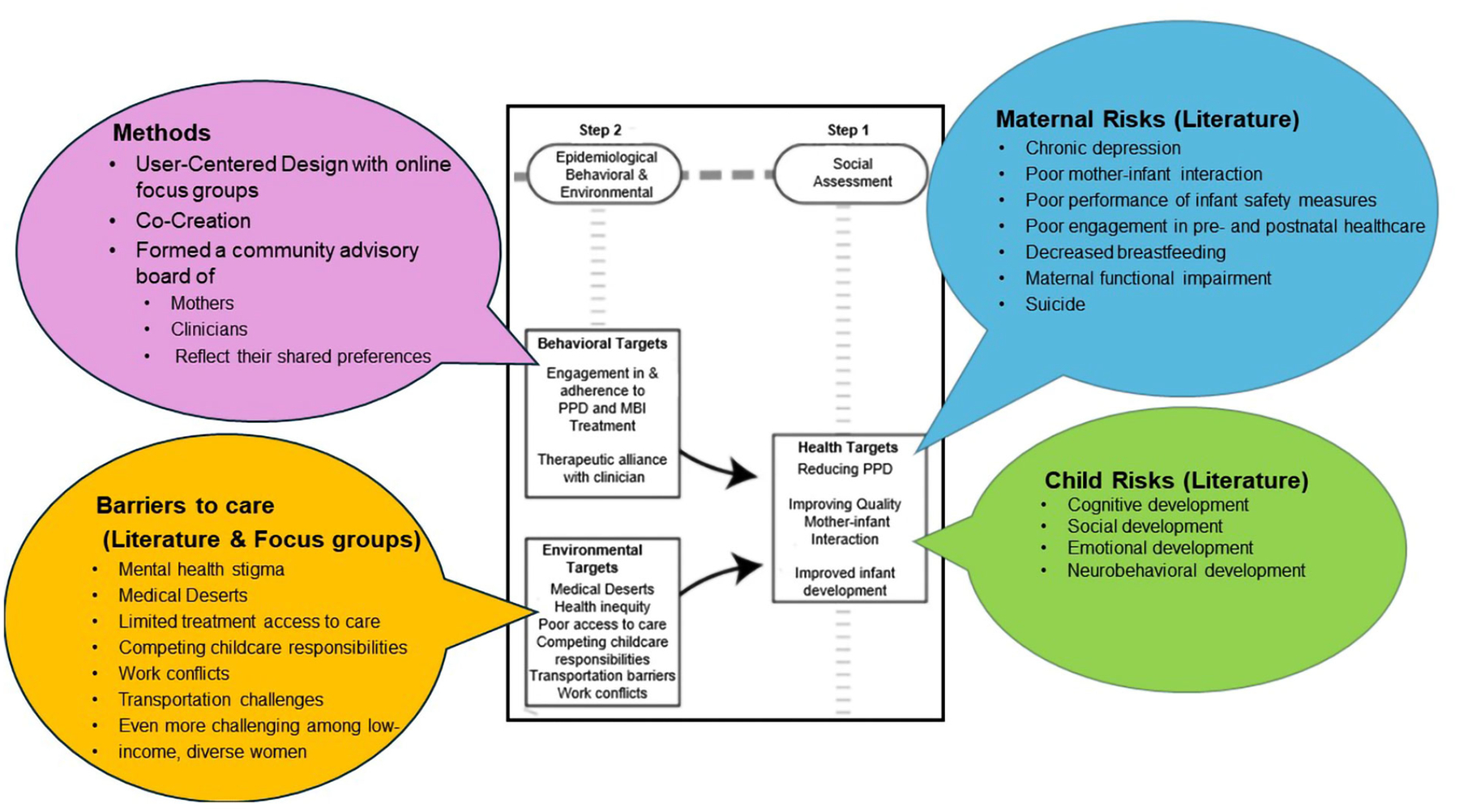
MommaConnect mapped onto steps 1 & 2 PRECEDE-PROCEED model [32] *Note*. Adapted with permission from “Development and evaluation of a youth mental health community awareness campaign-the compass strategy” by Wright A, McGorry PD, Harris MG, Jorm AF, Pennell K. BMC Public Health. 2006;6:215 (https://doi.org/10.1186/1471-2458-6-215). CC BY.

**Figure 3.**
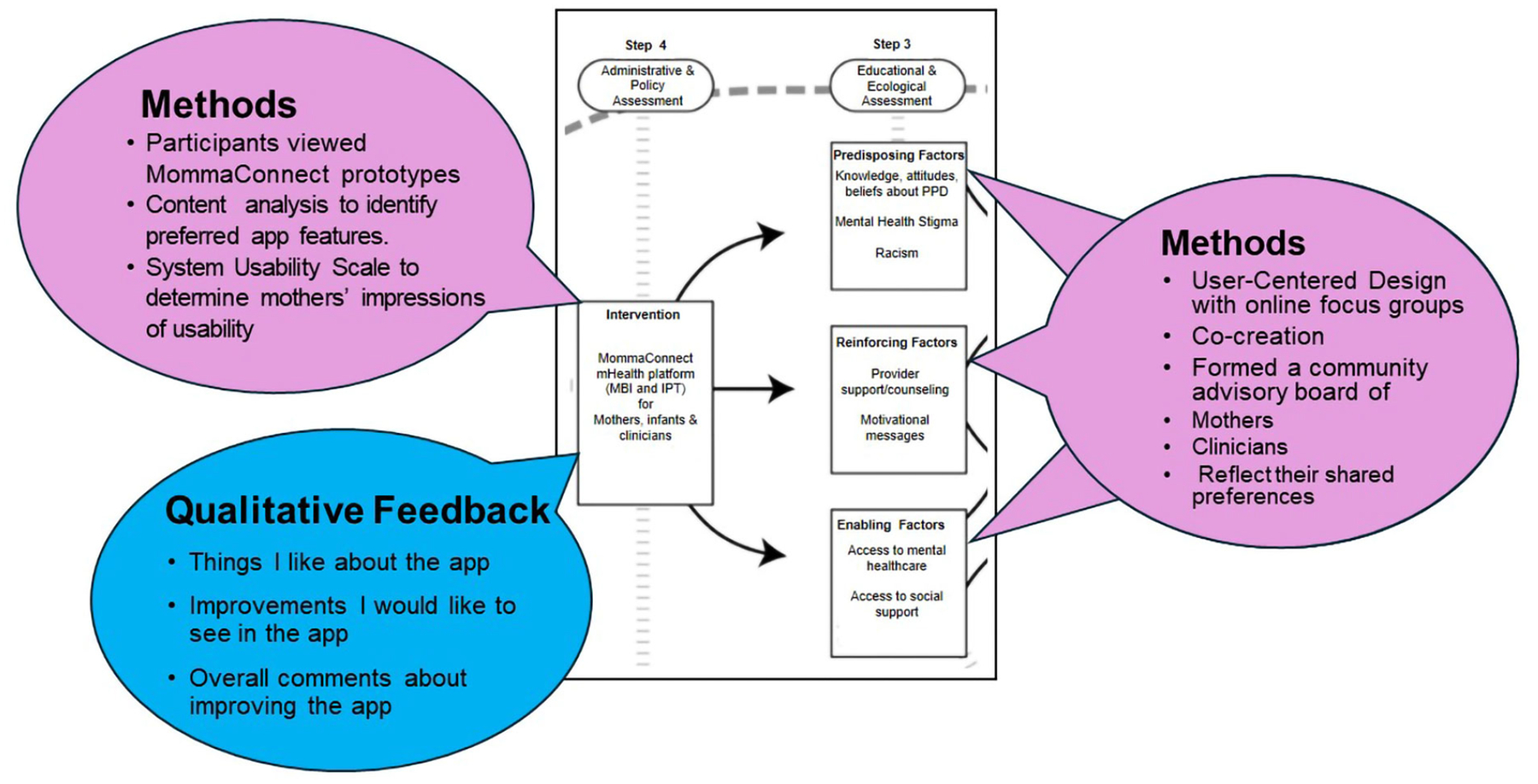
MommaConnect mapped onto steps 3 & 4 PRECEDE-PROCEED model [32] *Note*. Adapted with permission from “Development and evaluation of a youth mental health community awareness campaign-the compass strategy” by Wright A, McGorry PD, Harris MG, Jorm AF, Pennell K. BMC Public Health. 2006;6:215 (https://doi.org/10.1186/1471-2458-6-215). CC BY.

Guided by the PRECEDE-PROCEED model (Figure 1), we conducted formative research to begin relevant social, epidemiological, behavioral, environmental, educational, and ecological assessments consistent with steps 1–4 (Figures 2 and 3).

We incorporated the findings from our MBC program in steps 1–4 as well as relevant literature to inform these assessments. In addition, we assembled a community advisory board (CAB) of former MBC patients, who had experienced PPD, and perinatal mental health clinicians to conduct focus groups and in-depth interviews, respectively. These activities informed the preliminary design using personas, storyboards, journey maps of the users’ experiences, and interactive wireframes, as best practices in digital healthcare platform development. During the focus groups and interviews, we presented the design of content and app features to allow mothers with PPD and clinicians to interact with the MommaConnect digital healthcare platform. The total mean scores based on the System Usability Scale [35, 36] were 80 on the first round and 91 on the second. These scores are considered excellent in terms of usability, suggesting ease of use among both mothers and clinicians on the MommaConnect design and features. A score of 80.3 is equivalent to getting an A (the top 10% of scores) [37]. This is also the point where users are more likely to recommend the product to a friend.

During the focus groups, we also elicited the perspectives of mothers in the CAB regarding mental health treatment access, social determinants of health and health inequities that impact treatment, and how best to address their concerns. Specifically, we asked mothers about their current knowledge of PPD, experience of social determinants of health and health inequities, and barriers to the use of MommaConnect. To gain deeper insights into the challenges of delivering mental health services and addressing PPD from the clinicians’ perspectives, we collected qualitative data on their professional experiences to identify potential barriers to MommaConnect adoption.

**Figure 4.**
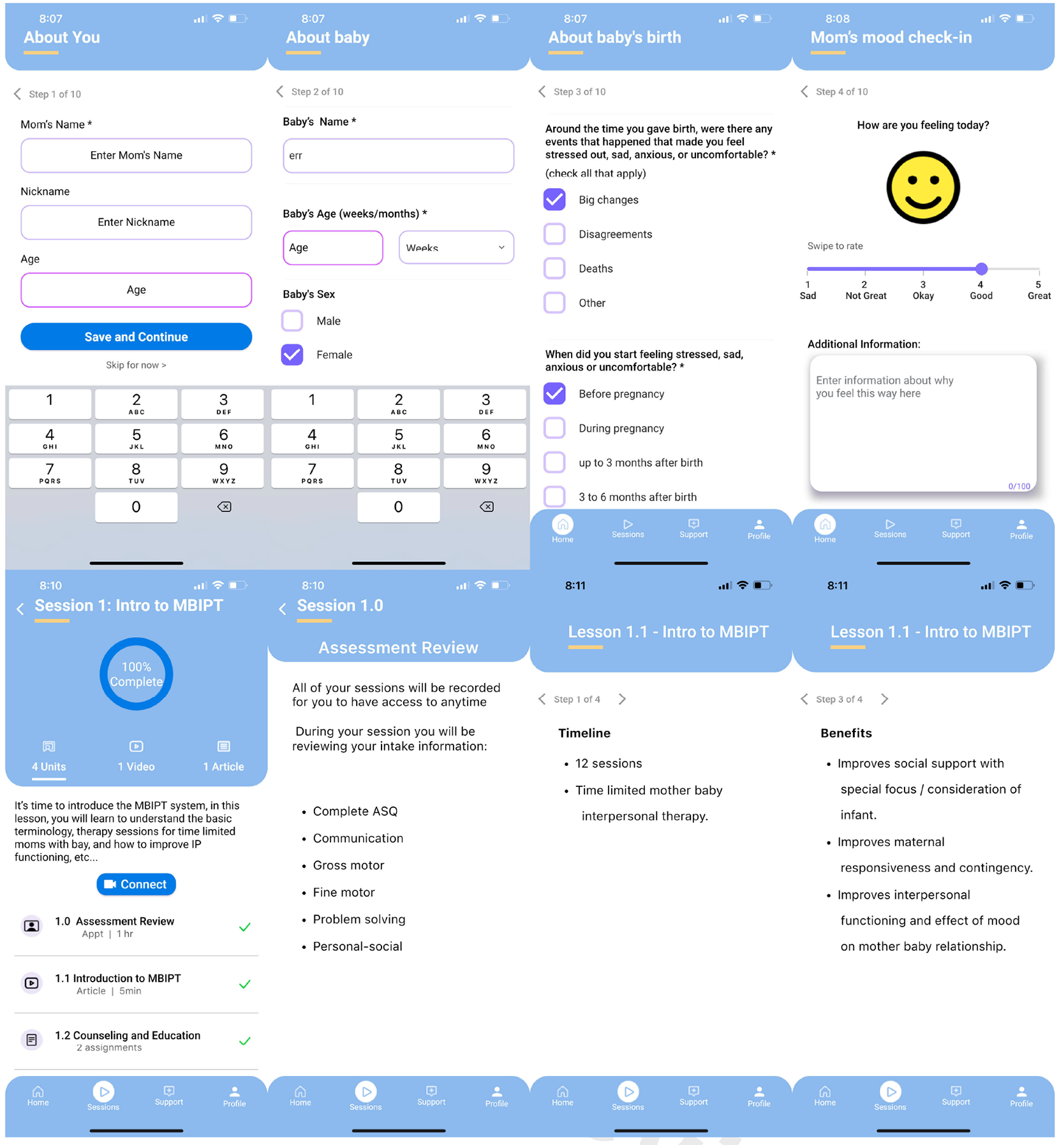
MommaConnect sample screenshots

As a result of focus group iterations exploring barriers and initial low-fidelity wireframes (Figure 4), we gathered essential feedback on challenges related to steps 2 and 3 of the model. For example, during our focus group, mothers mentioned they “did not know” they were experiencing depression or what depression entailed. In response, we created a micro-learning video as part of the app to provide information on recognizing depression and incorporated screeners to help identify symptoms. Additionally, mothers in the focus groups expressed a desire to retain information from the MBC in-person program, which was previously provided with paper “homework sheets” during individual sessions. Consequently, we designed the digital health platform to include access to prior therapy sessions that have been completed, as well as the ability to mark items for future reference. Other features validated by participants with a review of the low-fidelity wireframes include the ability to search for providers, conduct therapy sessions via Zoom-like sessions, and track moods. The current design and development of the MommaConnect platform include a comprehensive digital healthcare platform that provides a dashboard for mothers to complete the intake process, participate in sessions, and create their profiles. The platform also provides a clinician-facing portal to support virtual therapy sessions. A sample of some of the screens from the MommaConnect digital healthcare platform for mothers with PPD is provided in Figure 4.

## Expected results

Findings from focus groups with mothers and clinicians informed the research team of barriers and challenges to attending in-person therapy that will inform future work. Challenges included commuting to the MBC program or other treatment, symptom severity, and hectic home life. In addition, findings from the mothers’ focus groups and clinicians’ in-depth interviews also informed the research team about the desired features of our digital healthcare platform (Table 3).

Based on the findings from the focus groups and in-depth interviews, we developed preliminary culturally congruent MommaConnect content including PPD symptom assessment, information about PPD, psychotherapy content to address PPD symptoms, and Mother-Baby Interaction psychotherapy content to address deficits in the mother-infant relationship.

The research team also used Agile methodology [38] to develop MommaConnect iteratively using 4-week development cycles (i.e., sprints) to refine the platform for use on both Android and iOS mobile devices. MommaConnect uses the latest cross-platform technology, including ReactJS, MongoDB, and other technologies, to provide a robust platform with features such as video training, depression tracking, mother-infant attachment tracking, a dashboard, and communication tools between mothers and clinicians. At this point in our research, we are poised to evaluate the feasibility of MommaConnect through implementation including patient outcomes and process, impact, and outcome evaluation in Steps 5–8 of the PRECEDE-PROCEED model. Specifically, we will measure changes in depression with the Edinburgh Postnatal Depression Scale [39] and maternal-infant attachment with the Maternal Postnatal Attachment Scale [40] and assess system usability with the System Usability Scale [35, 36]. We hypothesize that the use of MommaConnect will reduce depression by 10 points, increase maternal-infant attachment by 6 points, and that system usability will be above 80 points. Future work also includes conducting a full-scale RCT to test efficacy and assessment of cost effectiveness and commercialization potential.

Despite growing treatment options, only 12–37% of women with PPD receive treatment, and many face significant barriers to treatment access, such as social determinants of health and health inequities [8–11, 19]. Remote treatment options, such as the MommaConnect digital healthcare platform, offer access for women across socio-economic strata who might not otherwise receive treatment. Without treatment, risks are high for poor maternal-child outcomes among women with PPD and their infants.

The PRECEDE-PROCEED model has been instrumental as a theoretical framework to guide the planning and iterative development of the MommaConnect digital healthcare platform by providing methods to identify social, epidemiological, behavioral, educational, ecological, organizational, and environmental barriers to implementation from women experiencing PPD. After completion of the assessment steps, PRECEDE-PROCEED will continue to guide the implementation and evaluation of MommaConnect that reflect the needs of women with PPD and their infants, and the preferences of the clinicians that provide perinatal mental health services. The next steps will involve additional feasibility testing, efficacy testing, and appraisal of cost-effectiveness and commercialization potential. The PRECEDE-PROCEED model provided a framework for a collection of preliminary evidence that suggests that MommaConnect holds promise to provide effective and culturally congruent delivery of maternal mental health services for women with PPD and their infants. Additionally, preliminary results suggest that MommaConnect has the potential to increase access to specialized PPD treatment, even for those in “medical deserts” and rural areas. In conclusion, based on our experience, we recommend the PRECEDE-PROCEED model as a comprehensive planning and theoretical framework for the development of other behavioral health interventions.

## Figures and Tables

**Table 1.** Mother Baby Connections (MBC) program demographics

Variable	N (%) unless otherwise noted (*N* = 80)
Age mean (SD)	31.1 (6.1)
Race	
White	28 (35.0%)
Black	32 (40.0%)
Hispanic	4 (5.0%)
Asian	5 (6.2%)
Other	11 (13.8%)
Annual income	
< $50,000	37 (46.3%)
$50,000-$100,000	16 (20.0%)
> $100,000	14 (17.6%)
Refused to answer	13 (16.3%)
Medical insurance	
Medicaid	42 (52.5%)
Commercial	30 (37.5%)
Self-pay	8 (10.0%)
Unemployed	44 (55.0%)
Education	
9–12th grade	3 (3.8%)
High school	14 (17.5%)
Attended college	18 (22.5%)
Associate degree	5 (6.3%)
Four-year college	20 (25.0%)
Attended graduate school	3 (3.8%)
Graduate school	16 (20.0%)
Other education	1 (1.0%)
Lives with partner	54 (67.5%)
Completed program	36 (45.0%)

**Table 2.** Mother Baby Connections (MBC) 6-year outcomes among women up to 1 year postpartum

Variable	*N*	Baseline		Exit		Mean Change		95% CI		*t*-test			Cohen’s d
		M	SD	M	SD	M_Δ_	SD	Lwr	Uppr	t	df	*p*	
Depression	37	17.9	4.7	10.0	4.9	7.9	5.0	6.26	9.6	9.68	36	< 0.001	1.59
Maternal functioning	35	70.5	19.2	90.1	12.5	19.6	17.7	13.5	25.7	6.54	34	< 0.001	1.11
Dyadic adjustment.	34	37.6	12.7	41.4	11.2	3.8	9.2	0.6	7.0	2.41	33	< 0.05	0.41
Parenting stress	34	49.0	9.9	42.0	8.5	7.0	7.6	4.3	9.7	5.34	33	< 0.001	0.92
Perceived stress	35	33.0	7.2	23.0	6.9	10.0	6.6	7.7	12.3	8.92	34	< 0.001	1.51
Emotional regulation	34	112.1	23.2	85.9	21.7	26.3	20.0	19.3	33.2	7.65	33	< 0.001	1.31
Birth Trauma	35	36.0	12.3	23.9	17.1	12.1	14.2	7.2	17.0	5.05	34	< 0.001	0.85
Insomnia	34	11.8	5.7	8.5	6.8	3.3	6.4	1.05	5.5	2.99	33	< 0.05	0.51
Program satisfaction (out of 32)	NA	NA	NA	29.4	NA	NA	NA	NA	NA	NA	NA	NA	NA

NA: not applicable

**Table 3.** Focus group data: desired features in the MommaConnect digital health platform

Focus group question prompts	Desired features
What do you think of the content? Is MommaConnect content culturally relevant to you?	Needs more culturally congruent content.
How can the design of MommaConnect help support you in your relationships with family?	Needs the capability of storing information from therapy sessions (e.g., skills discussed in sessions, and assigned homework).
How do you think it can help you with PPD?	Needs mechanisms to track symptoms and progress in therapy. Needs ways to address mental health stigma within the digital healthcare platform.
What do you think of the design of MommaConnect? What do you like/dislike about the wireframes?	Needs functionality to schedule appointments and contact the provider between sessions, contact information for emergent needs, and forums to chat with other mothers experiencing PPD.
How do you think MommaConnect can help you handle challenges with taking care of your baby?	Needs psychoeducation content about PPD and infant development.

PPD: postpartum depression

## Data Availability

The raw data supporting the conclusions of this manuscript will be made available by the authors, without undue reservation, to any qualified researcher.

## Abbreviations

MBC: Mother Baby Connections
PPD: postpartum depression
RCT: randomized controlled trial
